# Redox‐Respo*n*sive Tellurium‐Bridged Covalent Organic Frameworks/PEG Composites for Targeted Therapy of Diabetic Cardiomyopathy

**DOI:** 10.1002/advs.202509298

**Published:** 2025-11-18

**Authors:** Jing Xue, Jialu Zhuang, Taotao Fan, Songtao Tang, Junjun Wang, Xiaohui Liu, Jian Liu, Jin Shi, Jiaji Li, Wanjun Li, Danyou Hu, Xiaoqian Zhang, Hua Wang, Guiyang Zhang

**Affiliations:** ^1^ Department of Pharmacology School of Pharmacy Anhui Medical University Hefei 230032 China; ^2^ Department of Oncology The First Affiliated Hospital of Anhui Medical University Inflammation and Immune‐Mediated Diseases Laboratory of Anhui Province Anhui Medical University Hefei 230032 China; ^3^ Department of Functional Experiment Center School of Basic Medical Sciences Anhui Medical University Hefei 230032 China; ^4^ Department of Endocrinology First Affiliated Hospital Anhui Medical University Hefei 230032 China; ^5^ College of Chemical and Pharmaceutical Engineering Hefei Normal University Hefei 230601 China

**Keywords:** covalent organic frameworks, diabetic cardiomyopathy, glycated hemoglobin, reactive oxygen species

## Abstract

Diabetic cardiomyopathy, a major complication of diabetes, is strongly associated with elevated levels of glycated hemoglobin (HbA1c) and reactive oxygen species (ROS). However, effective clinical strategies to simultaneously lower HbA1c and ROS levels remain elusive, primarily due to the lack of therapeutic agents that can efficiently and synergistically interact with both biological macromolecules and small reactive molecules. To tackle this challenge, a redox‐responsive tellurium‐bridged covalent organic framework (Te‐COF) is developed whose surface is functionalized with hydrazine‐bonded polyethylene glycol (PEG), yielding a series of three emissive Te‐COF@PEG nanocomposites with varying PEG molecular weights (Te‐COF@PEG, M_W_ = 600, 2000, 6000 Da). In vitro studies demonstrate that Te‐COF@PEG composites efficiently remove HbA1c and total glycated protein from the plasma of diabetic patients, significantly lowering blood glucose levels without affecting serum levels of total proteins, lipids, and apolipoproteins. Among the composites, Te‐COF@PEG2000 exhibits the most promising therapeutic effects in diabetic mouse and rabbit models, including a significant reduction in fasting blood glucose, HbA1c, and inflammatory factor levels. Importantly, Te‐COF@PEG2000 induces macrophage polarization towards M2 phenotype, inhibits cardiomyocyte apoptosis, scavenges excess ROS, and synergistically improves myocardial injury. This study unlocks the immense potential of COF@polymer nanocomposites as a multifunctional platform for targeted diabetic cardiomyopathy therapy.

## Graphical abstract

This study unlocks the immense potential of COF@polymer nanocomposites as a multifunctional therapeutic platform for targeted drug delivery in diabetic cardiomyopathy.

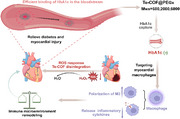

## 1 Introduction

Diabetic cardiomyopathies (DCM) are a serious complication of diabetes mellitus, characterized by structural and functional abnormalities of the myocardium that can ultimately lead to heart failure and death.^[^ ^1^, ^2^, ^3^ ^]^ Clinical studies have demonstrated that the incidence of DCM increases significantly as diabetes progresses, and cardiovascular complications will become the main cause of death in diabetic patients.^[^ ^4^ ^]^ In the diabetic state, chronic hyperglycemia leads to metabolic disorders in the body and impairs mitochondrial activity, resulting in large amounts of reactive oxygen species (ROS) and oxygen‐free radicals.^[^ ^5^ ^]^ These excessive ROS attack the cell membrane of cardiomyocytes and essential biomolecules such as nucleic acids, leading to increased apoptosis and necrosis of cardiomyocytes.^[^ ^6^ ^]^ Oxidative stress also alters the structure and function of intracellular proteins, impairing both contractile and diastolic function of cardiomyocytes and thereby exacerbating myocardial injury.^[^ ^7^ ^]^ In addition, ROS activate inflammatory signaling pathways such as nuclear factor‐κB and promote the expression and release of pro‐inflammatory cytokines.^[^ ^8^, ^9^, ^10^ ^]^ As a result, oxidative stress plays a crucial role in the pathogenesis of DCM, contributing to pathological changes including myocardial fibrosis and impaired cardiac function. Through its interaction with multiple pathways, oxidative stress profoundly disrupts cardiomyocyte structure and function.^[^ ^11^, ^12^ ^]^ Consequently, targeting oxidative stress presents a promising therapeutic strategy for the effective prevention and treatment of DCM.

Elevated glycated hemoglobin (HbA1c) is a key risk factor for cardiovascular complications in diabetes.^[^ ^13^, ^14^, ^15^, ^16^ ^]^ Current therapeutic strategies for diabetic cardiovascular disease mainly focus on regulating blood lipids and blood pressure based on glycemic control and delaying the progression of heart failure, which often yields suboptimal outcomes.^[^ ^17^, ^18^, ^19^, ^20^, ^21^ ^]^ In recent years, hydrazide enrichment of *N*‐glycopeptides has emerged as a viable strategy for glycoprotein detection.^[^ ^22^, ^23^ ^]^ This method leverages the specific chemical binding between hydrazide and glycan chains. Specifically, the cis‐diol moieties of the glycopeptide are oxidized to aldehydes, which then react with the hydrazide to form hydrazone (‐C═N‐NH‐), allowing for the selective capture and isolation of glycoprotein or glycan chains.^[^ ^24^, ^25^ ^]^ Under conditions of high HbA1c, glucose initially reacts with the *N*‐terminal valine residue of hemoglobin to form a reversible Schiff base (aldimine), which undergoes an Amadori rearrangement to yield a stable ketoamine structure.^[^ ^26^ ^]^ Notably, the hydrazide group (‐NHNH_2_) is highly reactive and can specifically form covalent hydrazone bonds (‐C═N‐NH‐) with either the keto group of HbA1c or the aldehyde group of the Schiff base. Hydrazide molecules exhibit high reactivity toward carbonyl groups, especially ketones, facilitating specific and stable hydrazone formation with HbA1c.^[^ ^27^, ^28^ ^]^ In contrast, normal hemoglobin (HbA) lacks such glycated modifications and does not contain free aldehyde or ketone groups, as its *β*‐chain N‐terminal valine remains unmodified. As such, hydrazide groups preferentially bind to HbA1c in the blood of hyperglycemic patients, with minimal interaction with unmodified HbA or other biomolecules lacking carbonyl functionalities. This inherent selectivity underpins the potential of hydrazide‐functionalized materials for the targeted removal or detection of HbA1c, offering a potent biochemical strategy to address diabetic complications at a molecular level.

Covalent organic frameworks (COFs), an emerging class of crystalline porous materials constructed from lightweight elements and linked via strong covalent bonds, have gained substantial attention in the biomedical field due to their compelling structural properties, such as exceptional structural tunability, high biocompatibility, large surface area, and ordered skeletons.^[^ ^29^, ^30^, ^31^, ^32^ ^]^ Unlike traditional porous materials, COFs possess inherent dynamic covalent bonds, which endow the frameworks with both structural stability under physiological conditions and responsiveness to endogenous stimuli such as pH changes, enzymatic activity, and redox environments.^[^ ^33^, ^34^ ^]^ Among dynamic moieties, disulfide bonds (‐S‐S‐) have been frequently exploited in controlled drug delivery due to their moderate bond energy (240 kJ mol^−1^) and sensitivity to both oxidative and reductive stimuli.^[^ ^35^, ^36^ ^]^ In our preliminary studies, a disulfide‐containing COF‐based drug‐carrying system demonstrated potent therapeutic efficacy with a single administration timed precisely at the ischemia‐reperfusion inflection point, outperforming the traditional continuous gavage drug delivery methods.^[^ ^37^ ^]^ Compared to disulfide bonds, tellurium bonds (‐Te‐Te‐) possess even greater redox sensitivity and have been employed in the design of low‐toxicity compounds such as AS101 and advanced redox‐responsive materials.^[^ ^38^, ^39^ ^]^ Capitalizing on the redox‐reversible nature of Te–Te bonds, the novel tellurium‐bridged COF offers adaptive regulation of ROS levels within the pathological microenvironment of DCM. Under oxidative stress, the tellurium bonds undergo specific oxidation reactions, neutralizing excess ROS by converting them into benign byproducts like water and oxygen via controlled electron transfer. Upon normalization of ROS levels, the framework restores its original chemical structure through reversible reduction reactions.^[^ ^40^ ^]^ This bidirectional “oxidation–reduction” adaptive response stems from the intrinsic redox sensitivity and chemical reversibility of dynamic covalent bonds, offering a promising strategy for real‐time ROS regulation and intelligent therapeutic intervention in redox‐related diseases such as DCM.

**Scheme 1:**
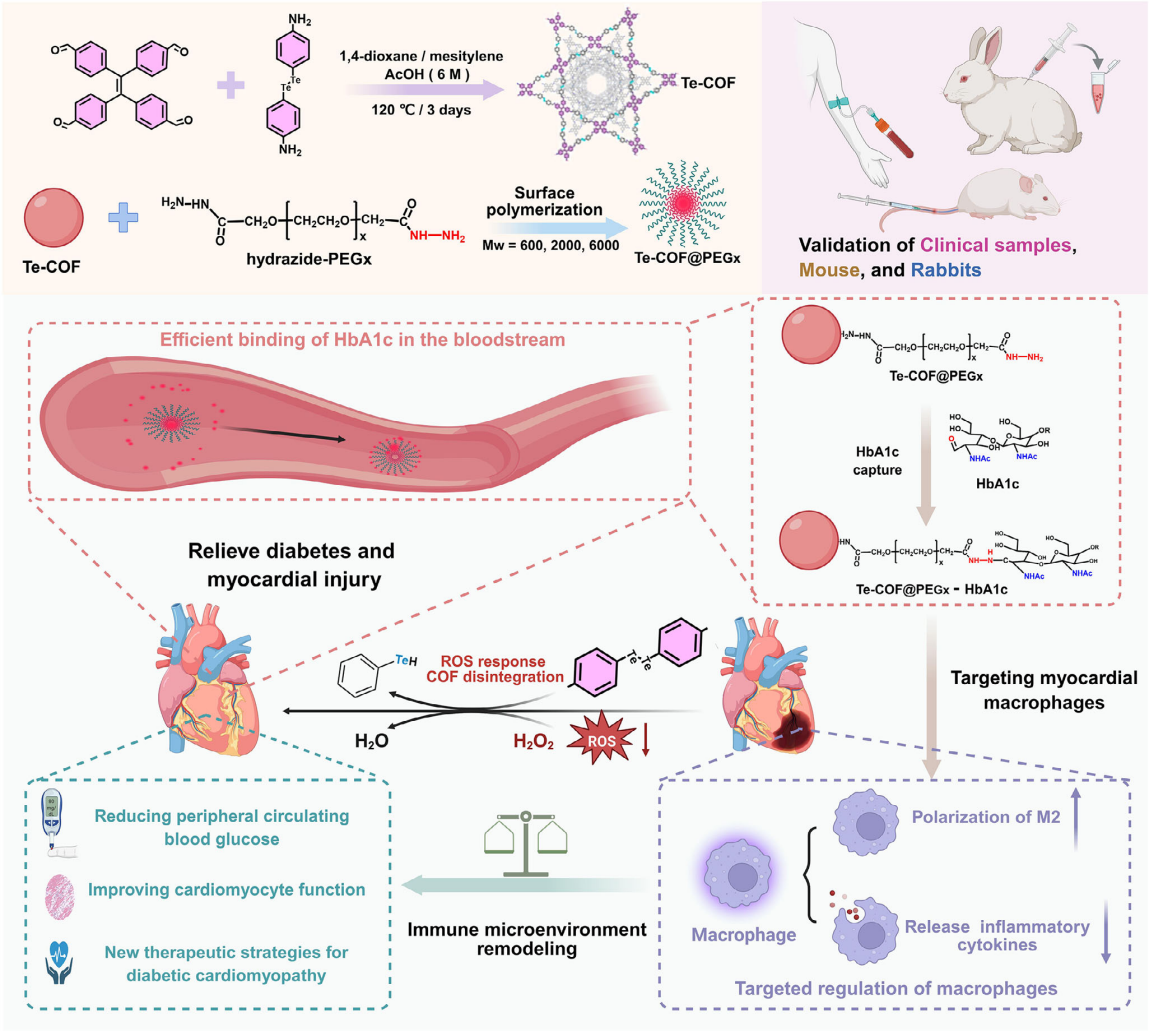
Schematic illustration of the synthesis of Te‐COF@polymer composites and mechanistic investigation of the simultaneous capture of HbA1c and reactive oxygen species for targeted therapy of diabetic cardiomyopathy.

In this study, we developed a tellurium‐bridged COF (termed Te‐COF), and subsequently grafted polyethylene glycol (PEG) containing hydrazide groups onto its surface to construct three COF@polymer composites (denoted as Te‐COF@PEG600, Te‐COF@PEG2000, and Te‐COF@PEG6000) (**Scheme** **1**). These hybrid materials were engineered to exhibit dual functionality: specific adsorption of HbA1c via hydrazide‐keto interactions and redox‐responsive scavenging of ROS via dynamic tellurium bonding. To evaluate their efficacy, we employed blood samples from diabetic patients to assess HbA1c and ROS capture. Among the three composites, Te‐COF@PEG2000 demonstrated excellent biocompatibility and efficient macrophage uptake. In both diabetic mouse and rabbit models, treatment with Te‐COF@PEG2000 led to significant reductions in fasting blood glucose, total glycated proteins, and HbA1c levels, along with marked improvements in inflammation, myocardial injury, cardiac remodeling, and ventricular wall thickness. Furthermore, RNA sequencing analysis showed that Te‐COF@PEG2000 modulated 288 differentially expressed genes, prominently suppressing the NF‐κB signaling pathway and associated immune‐inflammatory genes, thereby reversing myocardial fibrosis and structural deterioration characteristic of DCM. This study establishes a facile approach for the synthesis of Te‐COF@PEG composites for targeted therapy of DCM.

## 2 Results and Discussion

### 2.1 Synthesis and Characterization of Te‐COF and Te‐COF@PEG Composite

**Figure 1:**
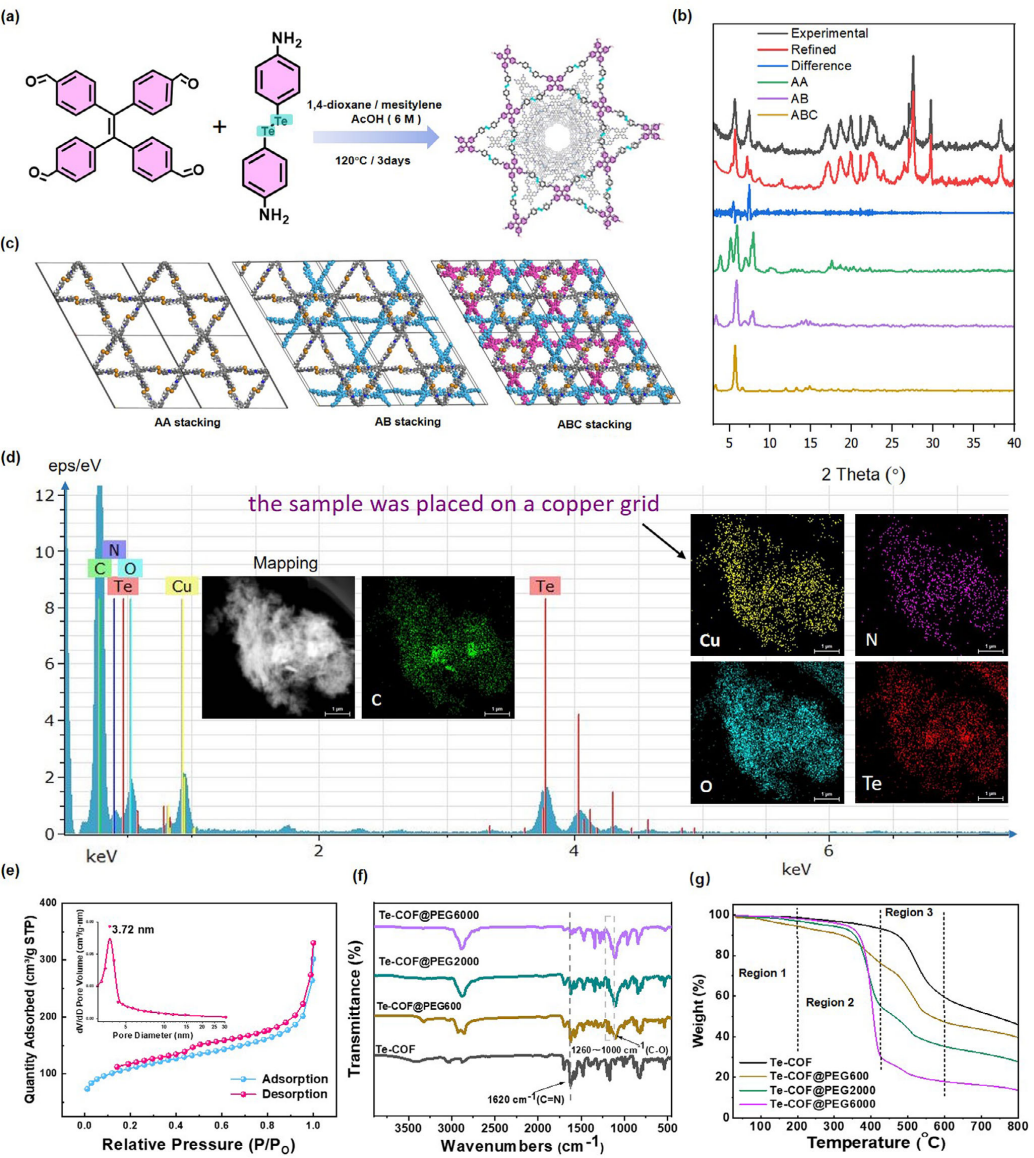
Structure and characterization of Te‐COF. a) Synthesis of Te‐COF. b) PXRD patterns of Te‐COF, with experimental data (black), Pawley refined data (red), and the difference between experimental and refined data. c) Space‐filling model of Te‐COF. d) HAADF‐STEM image (scale bar: 1 µm), elemental mapping of Te‐COF. e) N_2_ adsorption–desorption isotherm and the corresponding pore size distribution of Te‐COF. f) FT‐IR spectra of Te‐COF and Te‐COF@PEG composites. g) Thermogravimetric analysis of Te‐COF and Te‐COF@PEG under N_2_.

Ditelluride bonds (‐Te‐Te‐) have garnered significant attention in biomedical applications due to their low bond dissociation energy and dual redox‐responsive behavior, particularly in addressing oxidative stress within inflammatory microenvironments.^[^ ^40^ ^]^ Despite their promise, ditelluride moieties have not yet been incorporated into COFs thus far. In this work, we synthesized the first tellurium‐bridged COF (Te‐COF) through the Schiff‐base condensation of fluorescence‐traceable 4,4′,4′“,4′”'‐(ethene‐1,1,2,2‐tetrayl)tetrabenzaldehyde and ditelluride‐containing 4,4′‐ditellurodiphenylamine (**Figure** **1**a). The powder X‐ray diffraction (PXRD) pattern of the resulting Te‐COF exhibited a sharp diffraction peak at 5.35°, indicating the high crystallinity (Figure 1b). Pawley refinement of the PXRD data against the experimental pattern yielded good agreement factors (Rwp = 7.44%, Rp = 5.31%) (Figure S1, Supporting Information). To further validate the structure, three stacking modes (AA, AB, and ABC) were simulated using a hexagonal lattice (Figure 1c). The best fit was obtained with a single‐pore AB stacking model, whose Pawley‐refined PXRD pattern (blue curve) closely matched the experimental pattern (black curve). High‐angle annular dark‐field scanning transmission electron microscopy (HAADF‐STEM) combined with elemental mapping demonstrates the homogeneous distribution of C, N, O, and Te throughout the framework in Te‐COF (Figure 1d). Solid‐state ^13^C CP‐MAS NMR spectroscopy verified successful polymerization, with a key resonance at 155.6 ppm attributed to imine (C═N) bond formation (Figure S2, Supporting Information). The Nitrogen adsorption–desorption isotherm at 77 K confirmed the permanent porosity of Te‐COF with a Brunauer–Emmett–Teller (BET) surface area of 362 m^2^ g^−1^. Fitting the isotherm with the nonlocal density functional theory (NLDFT) model resulted in a pore size distribution centered at 3.72 nm, consistent with the predicted model (Figure 1e).

To enhance the bioactivity and medical applicability of Te‐COF, we functionalized it with hydrazide‐terminated PEG of varying molecular weights (600, 2000, and 6000 kDa) through reflux‐precipitation polymerization, giving rise to an HbA1c‐targeting nanoplatform (Scheme 1). The resulting PEGylated composites, designated Te‐COF@PEG, were thoroughly characterized by various analytical techniques. Fourier transform infrared (FT‐IR) spectra revealed the characteristic peak at 1620 cm^−1^, corresponding to C═N stretch of the imine linkage in Te‐COF, and a broad band at 1000–1260 cm^−1^ attributed to C‐O stretch from PEG, confirming the successful grafting of PEG onto Te‐COF (Figure 1f). Thermogravimetric analysis (TGA) under N_2_ atmosphere demonstrated distinct thermal behaviors: pristine Te‐COF exhibited high thermal stability up to 480 °C with a total mass loss of 46%. In contrast, Te‐COF@PEG composites exhibited an earlier decomposition onset (≈350 °C) and completed degradation by ≈550 °C. Notably, Te‐COF@PEG with increasing PEG molecular weight (600, 2000, and 6000 KDa) displayed progressively higher mass losses of 57%, 68%, and 88%, respectively (Figure 1g). Surface charge analysis provided further evidence for PEG grafting. Zeta potential measurements showed a marked shift from −1.35 mV for pristine Te‐COF to −12.31, −8.18, and −5.34 mV for the PEG600, PEG2000, and PEG6000 composites, respectively (Figure S3, Supporting Information). This trend of molecular weight‐dependent charge attenuation indicates the increasing surface shielding effect of the PEG corona, further confirming the successful surface functionalization.

### 2.2 In vitro Optimization of Te‐COF@PEG Composites

**Figure 2:**
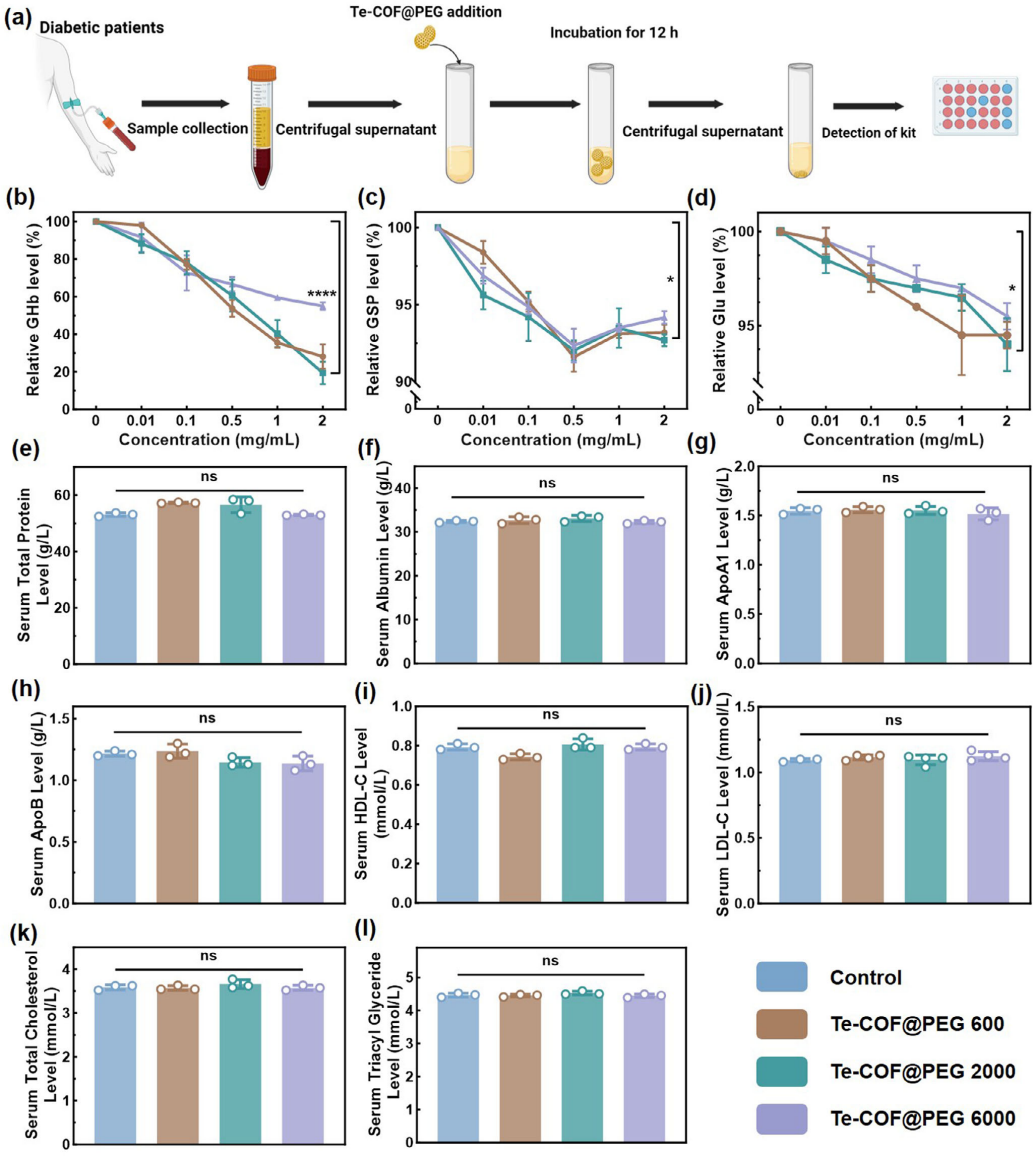
In vitro optimization of Te‐COF@PEG composites. a) Te‐COF@PEG composite was co‐incubated with the serum samples of diabetic patients, and changes in the relative levels of the total GSP b), glycosylated HbA1c c), blood Glu d), total protein e), ALB f), ApoA1 g), ApoB h), HDL‐C i), LDL‐C j), TC k) and TG l) were detected after the intervention of the Te‐COF@PEG composites. The values are mean ± SD (*n* = 3), and “ns” indicates no significant difference, demonstrating that the composite has no obvious adverse effects on these serum biomarkers at the tested concentrations*. p*‐Values: ^*^ *p* < 0.05 and ^****^ *p* < 0.0001.

To evaluate the efficacy of the Te‐COF@PEG in removing HbA1c, plasma and blood samples were collected from patients with diabetic myocardial ischemia in compliance with medical ethics. The bioactivity of the system was assessed using both static and dynamic incubation models. As shown in **Figure** **2**a, samples were incubated at 37 °C for 12 h with different Te‐COF@PEG composites at equal concentrations. The changes in total glycated protein, HbA1c, and blood glucose levels were determined by the nitrotetrazolium blue (NBT) method, thiobarbituric acid (TBA) method, and hexokinase method, respectively (Figure 2b–d). All five Te‐COF@PEG samples (PEG600, PEG2000, PEG3400, PEG5000, and PEG6000) significantly reduced the total glycated protein levels in high‐glucose plasma. Among them, the Te‐COF@PEG2000 group exhibited the highest HbA1c clearance efficiency (clearance > 90%). Interestingly, while the Te‐COF@PEG was primarily designed to target glycated proteins, a modest glucose‐lowering effect was also observed across all variants (Figure S4a–c, Supporting Information). This hypoglycemic activity may arise from a synergistic clearance mechanism involving the disruption of glycated protein‐glucose complexes.

**Figure 3:**
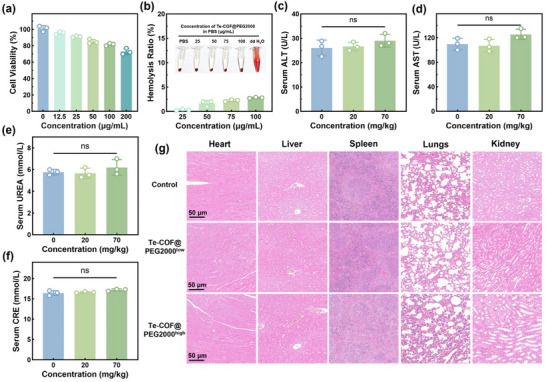
Biosafety evaluation of Te‐COF@PEG2000. a) MTT assay for myocardial macrophage proliferation. b) Hemolysis of Te‐COF@PEG2000 at different concentrations. Healthy RAW264.7 mice were injected with low and high doses of Te‐COF@PEG2000 solutions through the tail vein, and c) ALT, d) AST, e) UREA, and f) CRE were measured after 24 h. g) H&E staining of heart, liver, spleen, lung, and kidney tissues (Scale bar = 50 µm). Data represent mean ± SD (*n* = 3), and “ns” indicates no significant difference.

Biosafety evaluations confirmed that all five Te‐COF@PEG composites were effective in removing HbA1c without significantly affecting serum total protein (TP), albumin (ALB) or lipid metabolism markers, including apolipoprotein A1 (ApoA1), apolipoprotein B (ApoB), high‐density lipoprotein cholesterol (HDL‐C), low‐density lipoprotein cholesterol (LDL‐C), total cholesterol (TC) and triglyceride (TG) (Figure 2e–l, Figures S4d–k and S5, Supporting Information). Moreover, LDL‐C, TC, and TG were not significantly affected, further confirming the excellent biosafety of the systems. Given the superior performance of Te‐COF@PEG2000, it was selected for extended biocompatibility studies. MTT assays of Te‐COF@PEG2000 demonstrated no cytotoxicity toward RAW264.7 macrophages, even at a high concentration of 100 µg mL^−1^ (**Figure** **3**a). Hemolysis tests confirmed that Te‐COF@PEG2000 did not induce erythrocyte rupture within the therapeutic concentration range (Figure 3b). In vivo toxicity studies in mice, involving a high‐dose intravenous injection (70 mg kg^−1^), showed normal liver and kidney function based on alanine aminotransferase (ALT), aspartate aminotransferase (AST), urea (UREA), and creatinine (CRE) levels (Figure 3c–f). Histopathological examination revealed no abnormalities in major organs (Figure 3g). However, accumulation of Te‐COF@PEG2000 particles was observed in splenic tissues, which is likely due to the physiological properties of abundant macrophages in the spleen. Therefore, 20 mg kg^−1^ was chosen as a safe administration dose for subsequent in vivo experiments. Collectively, these experiments established a comprehensive safety profile from in vitro to in vivo. Notably, Te‐COF@PEG2000 selectively removed HbA1c without perturbing physiological parameters, positioning it as a compelling nanoplatform for treating diabetic complications.

### 2.3 In vivo Pharmacodynamic Evaluation of Te‐COF@PEG2000 in Mice

**Figure 4:**
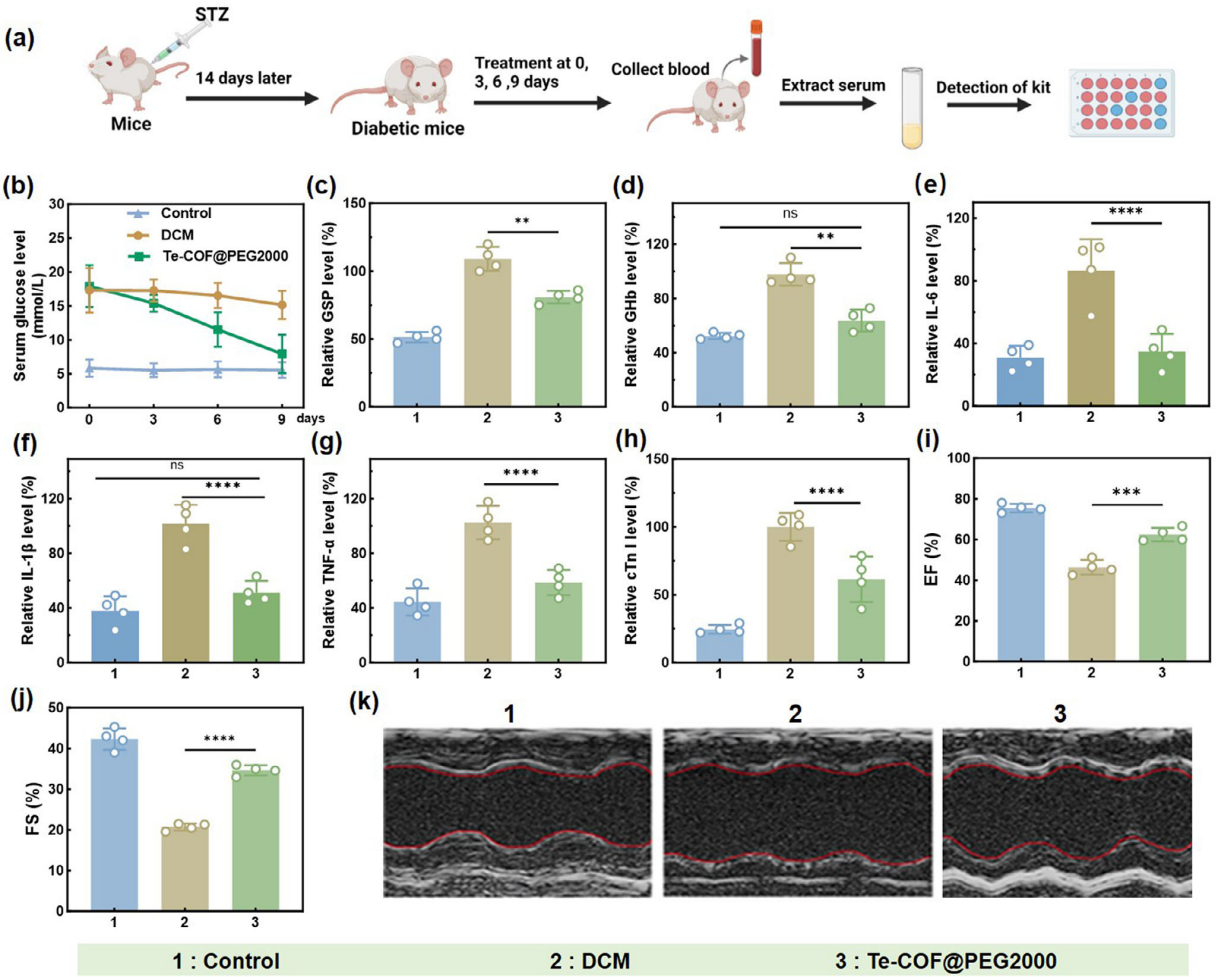
In vivo pharmacodynamic evaluation of Te‐COF@PEG2000 in mice. a) Schematic diagram of the experimental method after establishing the STZ‐induced diabetic mouse model. b) Dynamic monitoring of blood glucose levels in each group at days 0, 3, 6, and 9 post‐injection of Te‐COF@PEG2000 with the same concentration. At the end of the experiment, the serum of mice was used to detect GSP c) and HbA1c d), and IL‐6 e), IL‐1β f), TNF‐α g), and other inflammatory factors and myocardial injury marker cTnI h). k) Representative M‐mode images from echocardiography at the end of the experiment. EF% i) and FS% j) were measured to evaluate mouse cardiac function. 1: Control group, 2: DCM group, 3: Te‐COF@PEG2000 group. Data represent mean ± SD (*n* = 4), *p*‐Values: ^**^ *p* < 0.01 and ^****^ *p* < 0.0001.

To assess the in vivo therapeutic efficacy of Te‐COF@PEG2000, we constructed a streptozotocin (STZ)‐induced diabetic mouse model (persistent fasting blood glucose > 16.7 mmol L^−1^).^[^ ^41^ ^]^ Mice were randomly assigned in a double‐blind manner to three groups: a treatment group receiving Te‐COF@PEG2000 (20 mg kg^−1^), a diabetic cardiomyopathy model group (DCM group), and a healthy control group (**Figure** **4**a). Ambulatory glucose monitoring revealed that Te‐COF@PEG2000 exhibited a more pronounced glucose‐lowering effect in vivo compared to in vitro (Figure 4b). Consistent with prior findings, glycated serum protein (GSP, NBT method) and glycated hemoglobin (GHb, TBA method) levels were significantly reduced in the treatment group (Figure 4c,d). Inflammatory markers (IL‐1β, IL‐6, TNF‐α) were markedly decreased following treatment, indicating attenuation of systemic inflammation (Figure 4e,g). Additionally, levels of cardiac troponin I (cTnI), a marker of myocardial injury,^[^ ^42^ ^]^ were significantly lowered after Te‐COF@PEG2000 treatment (Figure 4h), suggesting cardioprotective effects. Cardiac ultrasonography showed that mice in the DCM group developed cardiac remodeling features, such as left ventricular dilatation and myocardial hypertrophy, accompanied by significant reductions in ejection fraction (EF%) and fractional shortening (FS%). In contrast, the Te‐COF@PEG2000‐treated group exhibited substantial functional recovery, with EF% and FS% restored to ≈60% and 35%, respectively, which were close to normal physiological values (Figure 4i,j). The Te‐COF@PEG2000 group was further confirmed to improve cardiac remodeling and cardiac function in diabetic mice (Figure 4k). Taken together, these results demonstrate that Te‐COF@PEG2000 not only effectively lowers blood glucose and glycated protein levels in vivo but also mitigates inflammation, reduces myocardial injury, and improves cardiac remodeling and function in diabetic mice.

**Figure 5:**
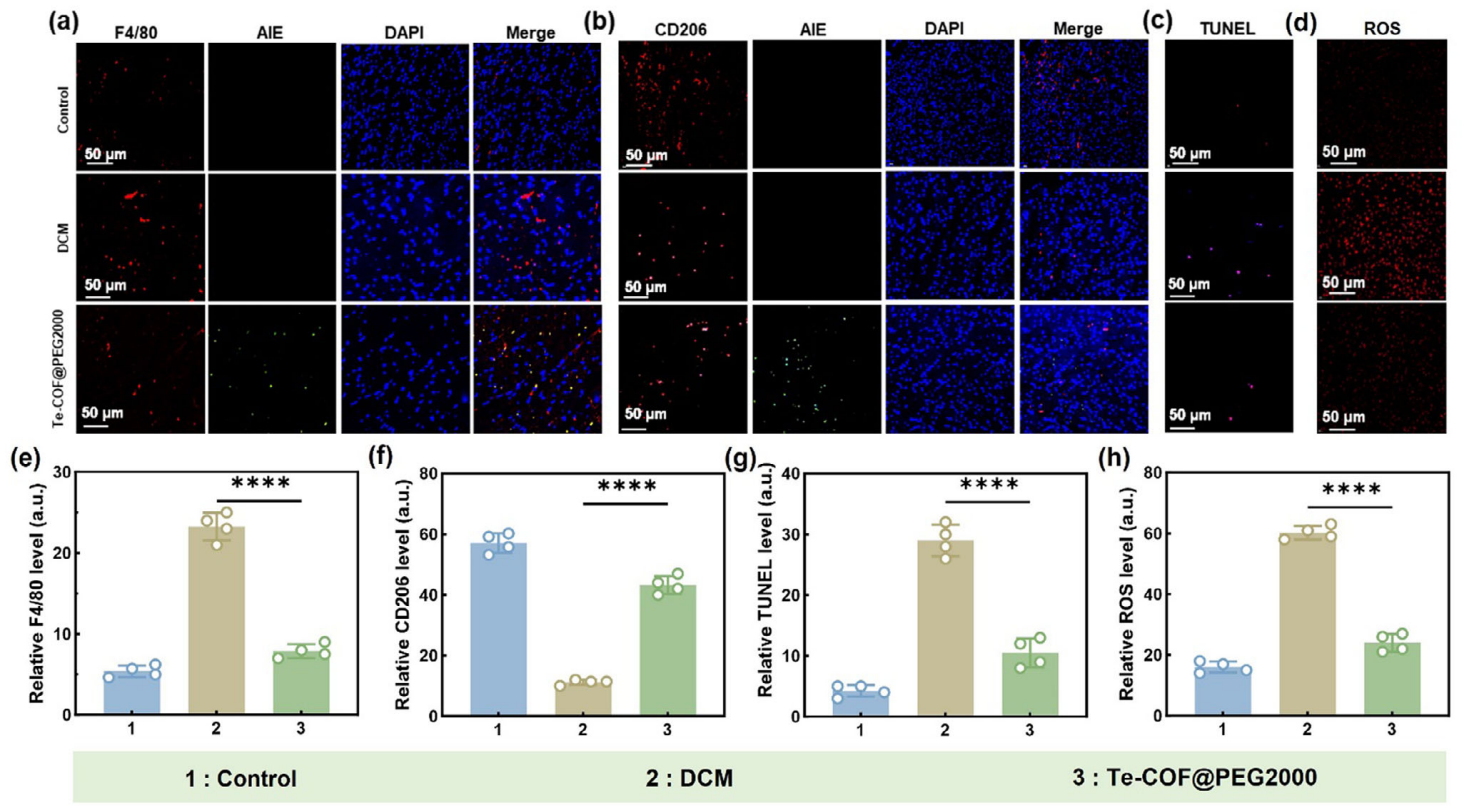
Fluorescent staining analysis of mouse myocardial tissue. a,e) F4/80 fluorescence staining of mice myocardial tissue and quantitative analysis of fluorescence intensity. b,f) CD206 fluorescence staining of mice myocardial tissue and quantitative analysis of fluorescence intensity. c,g) TUNEL fluorescence staining of mice myocardial tissue and quantitative analysis of fluorescence intensity. d,h) ROS staining of mice myocardial tissue and quantitative analysis of fluorescence intensity. Scale bar, 50 µm. Data represent mean ± SD (*n* = 4), *p*‐Values: ^****^ *p* < 0.0001.

Macrophages, as key regulators of the innate immune system, play a dual role in the pathogenesis of DCM.^[^ ^43^ ^]^ To assess the immunomodulatory effects of Te‐COF@PEG2000 on cardiac macrophages, we conducted immunofluorescence staining in myocardial tissues. Immunofluorescence staining for the macrophage marker F4/80 in myocardial tissue revealed that Te‐COF@PEG2000 treatment effectively reduced macrophage infiltration (**Figure** **5**a,e). Notably, strong colocalization of F4/80 (red) and Te‐COF@PEG2000 (green) fluorescence indicated targeted delivery of Te‐COF@PEG2000 to myocardial macrophages, suggesting immune regulatory effects. Moreover, CD206, a specific surface marker of M2‐type macrophages, reflected macrophage polarization from a pro‐inflammatory to an anti‐inflammatory phenotype when upregulated.^[^ ^44^ ^]^ Immunofluorescence analysis showed a significant increase in CD206 expression in the treatment group (Figure 5b,f), suggesting that Te‐COF@PEG2000 effectively promoted macrophage polarization toward an anti‐inflammatory phenotype. Consistent with these findings, TUNEL staining showed a significant reduction (*p* < 0.001) in the rate of cardiomyocyte apoptosis in the intervention group (10.5 ± 1.2%) compared to the model group (29 ± 2.8%) (Figure 5c,g). In addition, the dihydroethidium (DHE) fluorescent probe assay demonstrated a significant decrease in myocardial ROS levels to 24.8 ± 3.2% (compared to 59.4 ± 2.4% in the model group, *p* < 0.001) (Figure 5d,h), suggesting that Te‐COF@PEG2000 ameliorated myocardial injury by inhibiting oxidative stress and apoptosis.

**Figure 6:**
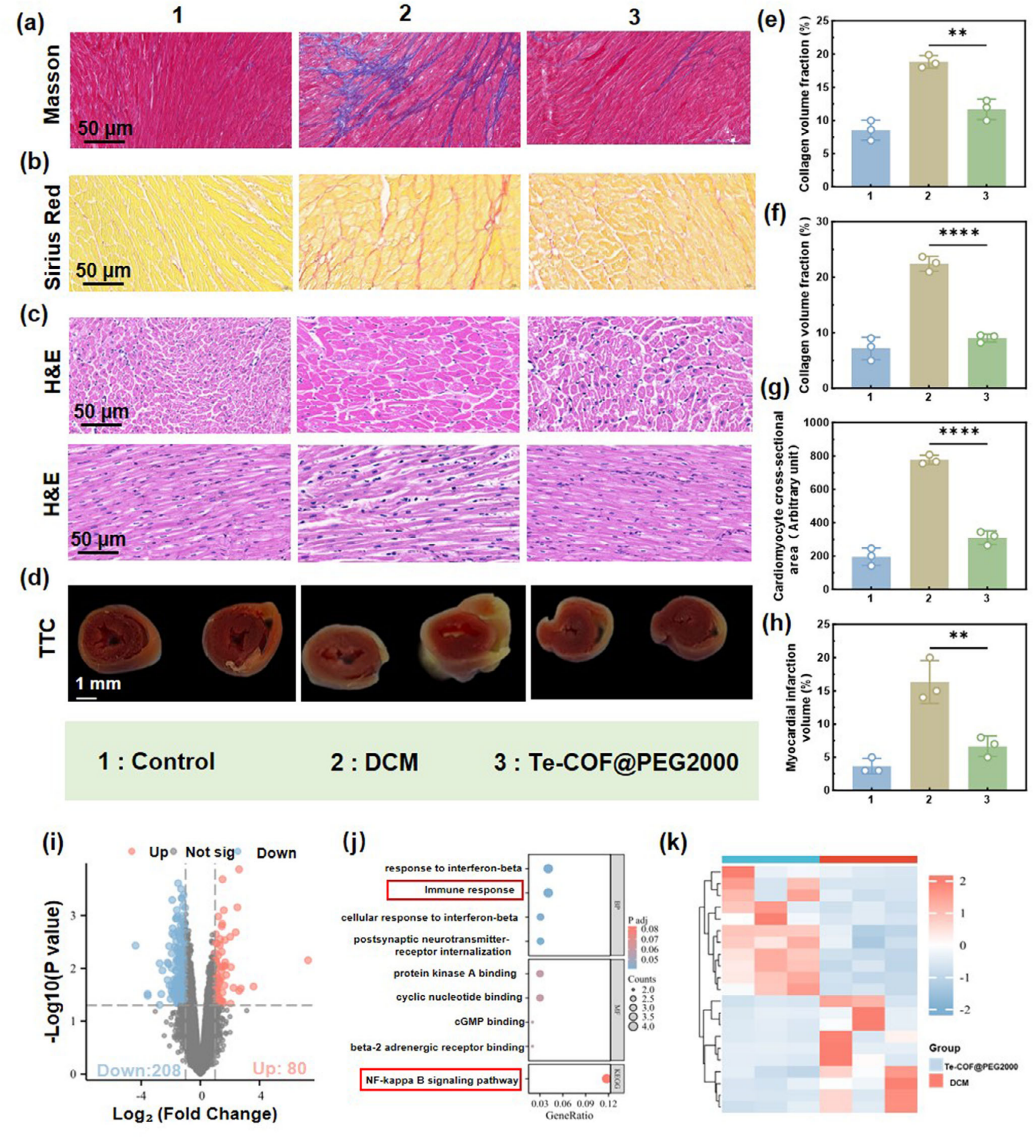
Te‐COF@PEG2000 treatment significantly improves cardiac function in mice. a) Masson staining images of the myocardial tissue of mice in each group, and e) quantification of the CVF of the myocardial tissue. Scale bar, 50 µm. b) Sirius red staining images of the myocardial tissue of mice in each group, and f) quantification of the CVF of the myocardial tissue. Scale bar, 50 µm. c) H&E staining images of the myocardial tissue of mice in each group, and g) quantification of the necrotic area of the myocardial tissue. Scale bar, 50 µm. h) Myocardial infarction volume was evaluated by d) TTC staining analysis. Scale bar, 1 mm. i) Volcano plot analysis of gene expression differences between the Te‐COF@PEG2000 intervention group and the DCM group. j) GO enrichment analysis maps on cellular components, molecular functions, and differential gene expression in biological processes, and KEGG pathway enrichment analysis of the differential genes. k) Heatmap analysis of transcriptional expression profiles after Te‐COF@PEG2000 treatment. Data represent mean ± SD (*n* = 3), *p*‐Values: ^**^ *p* < 0.01, ^****^ *p* < 0.0001.

Consistent with the in vitro results, histopathological analysis of the intervention mice exhibited significantly reduced myocardial fibrosis (Masson and Sirius red staining), collagen deposition (CVF), cardiomyocyte hypertrophy (H&E staining), and infarct size (TTC staining) compared to the DCM group (**Figure** **6**a–h). In line with these structural improvements, RNA sequencing (RNA‐seq) identified 288 differentially expressed genes, including 80 upregulated and 208 downregulated. KEGG pathway analysis indicated that these transcriptional changes were closely associated with the regulation of immune‐inflammatory responses and significant inhibition of NF‐κB pathway activity (Figure 6i–k). Collectively, our study underscores the vast potential of Te‐COF@PEG2000 as an effective therapeutic agent for DCM therapy.

### 2.4 Evaluation of Diabetic Cardiomyopathy using Rabbit Models

**Figure 7:**
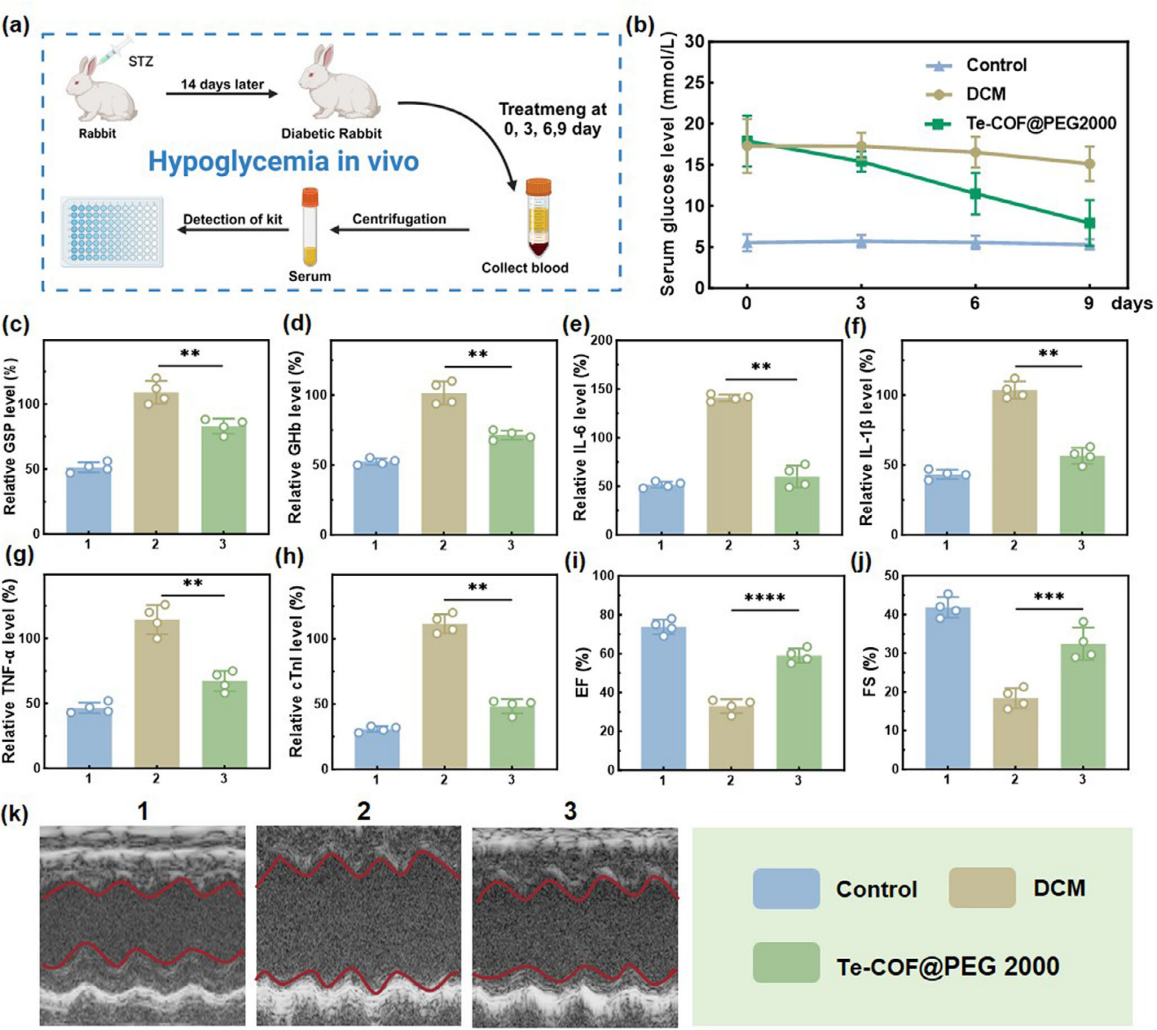
In Vivo pharmacodynamic evaluation of Te‐COF@PEG2000 in rabbits. a) Schematic diagram of the experimental method after establishing the STZ‐induced diabetic rabbits’ model. b) Dynamic monitoring of blood glucose levels in each group at days 0, 3, 6, and 9 post‐injection of Te‐COF@PEG2000 with the same concentration. At the end of the experiment, the serum of rabbits was used to detect GSP c) and HbA1c d), and IL‐6 e), IL‐1β f), TNF‐α g), and other inflammatory factors and myocardial injury marker cTnI h). k) Representative M‐mode images from echocardiography at the end of the experiment. EF% i) and FS% j) were measured to evaluate rabbits’ cardiac function. Data represent mean ± SD (*n* = 4), ^**^ *p < 0.01*, ^***^ *p < 0.001*, ^****^ *p* < *0.0001*.

To further validate the therapeutic effects of Te‐COF@PEG2000 on DCM, we employed a rabbit model of DCM, which offers greater anatomical and metabolic similarities to human cardiac physiology compared to murine models, thus allowing for more precise assessment of dynamic cardiac function. As depicted in **Figure** **7**a, DCM was induced in rabbits using STZ. Consistent with our murine data, Te‐COF@PEG2000 treatment significantly lowered levels of serum glucose (Figure 7b), total glycated proteins (Figure 7c), HbA1c (Figure 7d), inflammatory cytokines such as IL‐1β, IL‐6, and TNF‐α (Figure 7e–g), and the cardiac injury biomarker cTnI (Figure 7h). Echocardiographic analysis revealed significant improvements in myocardial function, with enhanced ejection fraction (EF%) and fractional shortening (FS%), along with normalization of myocardial thickness (Figure 7i–k).

**Figure 8:**
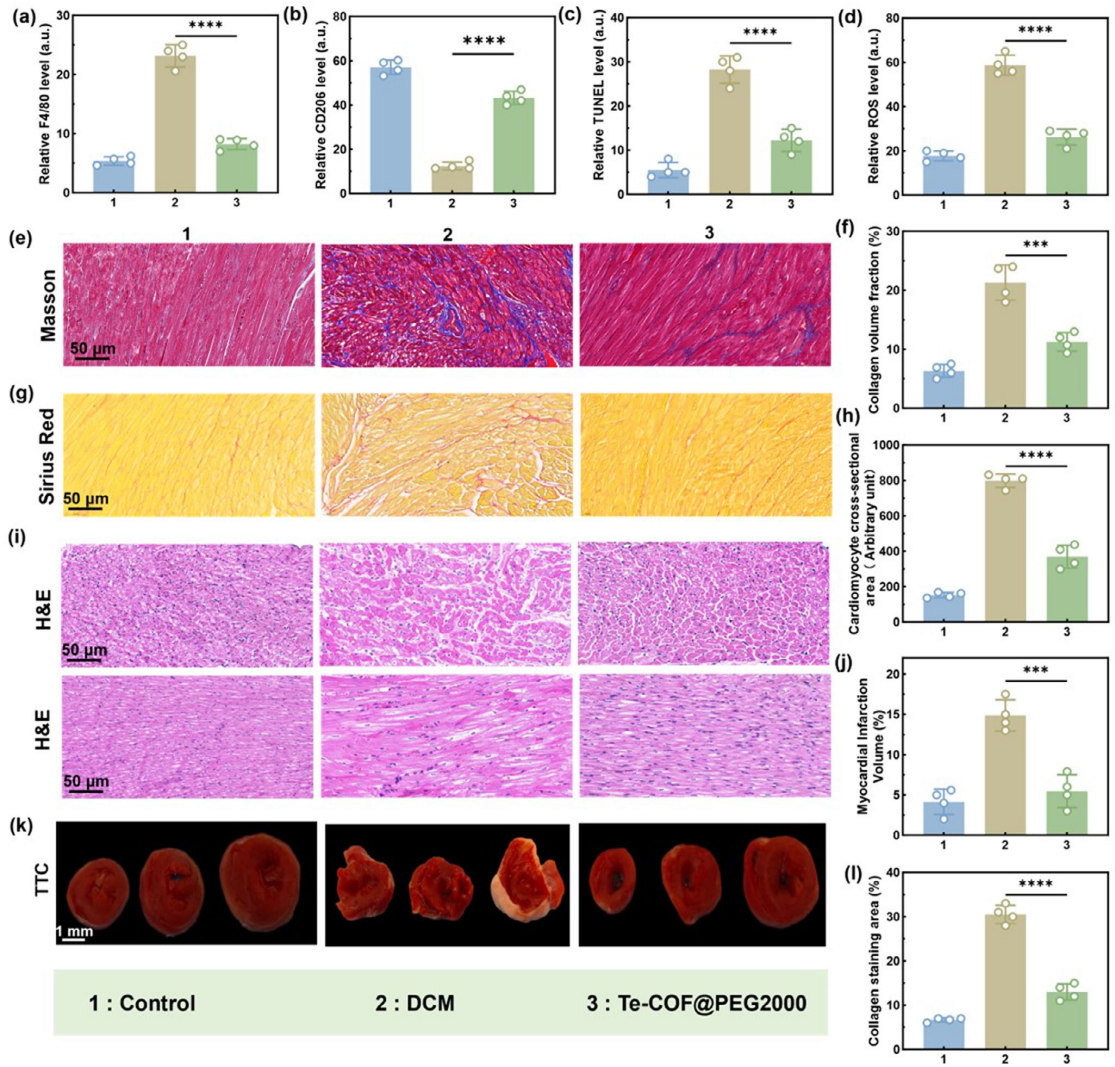
Te‐COF@PEG2000 treatment significantly improves cardiac function in diabetic rabbits. a) Quantification of F4/80 fluorescence staining of the rabbit's myocardial tissue. b) Quantification of CD206 fluorescence staining of the rabbit's myocardial tissue. c) Quantification of TUNEL fluorescence staining of the rabbit's myocardial tissue. d) Quantification of ROS fluorescence staining of the rabbit's myocardial tissue. e) Masson staining images of the myocardial tissue of rabbits in each group, and f) quantification of the CVF of the myocardial tissue. Scale bar, 50 µm. g) Sirius red staining images of the myocardial tissue of rabbits in each group, and h) quantification of the CVF of the myocardial tissue. Scale bar, 50 µm. i) H&E staining images of the myocardial tissue of rabbits in each group, and j) quantification of the necrotic area of the myocardial tissue. Scale bar, 50 µm. l) Myocardial infarction volume of rabbits was evaluated by k) TTC staining analysis. Scale bar, 1 mm. Data represent mean ± SD (*n* = 4), *p*‐Values: ^**^ *p* < 0.01, ^***^ *p* < 0.001, ^****^ *p* < 0.0001.

Immunofluorescence staining further demonstrated reduced macrophage infiltration, polarization toward the anti‐inflammatory M2 phenotype, diminished cardiomyocyte apoptosis, and alleviation of oxidative stress via decreased ROS levels in cardiac tissue (**Figure** **8**a–d; Figures S6–S9, Supporting Information). In addition, histopathological evaluations via Masson's trichrome and Sirius Red staining confirmed a significant reduction in myocardial fibrosis and collagen deposition (Figure 8e–h), while H&E staining showed reduced cardiomyocyte cross‐sectional area and restored cellular morphology (Figure 8i,g). Importantly, TTC staining further validated the infarct size‐reducing effects of Te‐COF@PEG2000 (Figure 8k,l). Overall, these findings validate the therapeutic potential of Te‐COF@PEG2000 in a large animal model through a multi‐modal mechanism of action, highlighting its prospects as a translational nanoplatform for the clinical management of DCM.

## 3 Discussion

In this work, a highly emissive tellurium‐bridged COF was synthesized by co‐polymerization of tetrastyrene‐based aldehydes and tellurium‐bridged amines, followed by PEG surface modification. The resulting Te‐COF@PEG composites exhibit several unique advantages: 1) The tellurium bond energy (138 kJ mol^−1^) is significantly lower than that of the traditional disulfide bond (240 kJ mol^−1^), enabling faster redox reactions and allowing for a precise response to dynamic changes in ROS within the microenvironment of DCM. 2) Surface modification with PEG of varying molecular weights enhances the aqueous dispersion and blood compatibility, while also improving hydrazone compatibility through interaction with hydrazine groups. 3) It demonstrates high efficacy in capturing HbA1c due to the specific binding affinity between hydrazine and aldehyde groups, offering vast potential for treating DCM. 4) The aggregation‐induced luminescence (AIE) property of tetrastyrene moieties imparts real‐time imaging capability, enabling the tracking and visualization of cardiac tissue distribution and providing a valuable tool for assessing therapeutic outcomes.^[^ ^45^ ^]^ This multifunctional design overcomes the limitations of conventional nanomaterials with singular functions and offers a promising strategy for the treatment of diabetic complications.

Among the Te‐COF@PEG composites, Te‐COF@PEG2000 exhibits a unique dual scavenging mechanism against the two key pathological factors of DCM: hyperglycemia‐induced HbA1c accumulation and ROS overproduction. First, Te‐COF@PEG2000 significantly reduces plasma glycated protein levels in diabetic patients by specifically capturing HbA1c through hydrazide bonding. Second, it effectively scavenges ROS via glutathione peroxidase‐like activity of tellurium bonds. This synergistic effect interrupts the pathological cycle of hyperglycemia, advances glycosylation end‐products (AGEs), ROS, and inflammation, thereby addressing the root causes of oxidative stress and glycotoxicity on cardiomyocytes. Importantly, Te‐COF@PEG2000 shows minimal effect on normal blood components such as albumin and lipoproteins, suggesting excellent targeting specificity and biosafety. As shown in Figure S10 (Supporting Information), Te‐COF@PEG2000 exhibited pronounced liver accumulation at both 24 and 72 h. In contrast to other PEG molecular weights, this formulation also demonstrated superior stability and significant time‐dependent retention in the heart, as clearly visualized by its distinct fluorescence intensity profiles across the examined organs.

Mechanistic studies revealed that Te‐COF@PEG2000 effectively regulates macrophage polarization, reduces myocardial macrophage infiltration, and promotes the conversion of M1 pro‐inflammatory phenotype to M2 anti‐inflammatory phenotype. RNA sequencing analysis further showed that Te‐COF@PEG2000 inhibits the NF‐κB signaling pathway and downregulates the expression of pro‐inflammatory factors (IL‐1β, IL‐6, and TNF‐α). This immune reprogramming effectively attenuates cardiomyocyte apoptosis and fibrosis, ultimately leading to improved cardiac function, as evidenced by enhanced left ventricular ejection fraction and shortening fraction.

The cross‐species efficacy of Te‐COF@PEG2000 was validated using clinical samples and both mouse and rabbit models. The improved myocardial fibrosis and ventricular remodeling in rabbits further underscore the translational potential of this material, given that the rabbit's cardiac anatomy more closely resembles that of humans. In both animal models, Te‐COF@PEG2000 significantly reduced myocardial infarct size and mitigated metabolic disturbances, indicating that its therapeutic mechanism is broadly applicable. These findings establish Te‐COF@PEG2000 as a promising strategy for the precise treatment of DCM and lay a solid foundation for the development of next‐generation therapeutics targeting diabetic complications.

## 4 Conclusion

In summary, we have developed a redox‐responsive tellurium‐bridged COF assembled with hydrazide‐terminated PEG chains with different molecular weights. In vitro experiments using plasma from diabetic patients revealed that Te‐COF@PEG2000 effectively removed total glycated protein and HbA1c, significantly reduced blood glucose levels, exhibited no interference with serum total protein, lipid, and lipoprotein levels, and showed excellent biocompatibility. Notably, it exhibited strong myocardial targeting and prolonged retention. In diabetic mouse and rabbit models, Te‐COF@PEG2000 was highly effective in reducing fasting glucose, HbA1c, and inflammatory cytokine levels. Echocardiographic analysis confirmed reversal of pathological ventricular remodeling. Mechanistically, Te‐COF@PEG2000 synergistically ameliorated myocardial injury by promoting macrophage polarization toward the M2 phenotype, inhibiting cardiomyocyte apoptosis, and scavenging ROS. In addition, it also reduced CVF, restored cardiomyocyte cross‐sectional area, reduced infarct size, and ultimately reversed the structural deterioration of DCM. RNA sequencing further demonstrated that Te‐COF@PEG2000 regulated 288 differentially expressed genes, inhibited the activation of the NF‐κB pathway and immunoinflammatory genes, and reversed myocardial fibrosis. These findings, validated across species, highlight Te‐COF@PEG2000 as a promising candidate for clinical translation and a novel therapeutic strategy for metabolic cardiovascular complications in diabetes.

## 5 Experimental Section

### Ethical Approval

The animal study was approved by the Animal Ethics Committee of the authors’ institution (No.14–862). All experimental procedures were authorized by the Committee on the Ethics of Animal Experiments of Anhui Medical University. The clinical sample blood tests used in this study were also approved by the Ethical Review Committee (Approval No.: pj2023‐04‐10). Written informed consent was provided for all blood samples provided by patients/participants to participate in this study. For the 20 patients with diabetic myocardial ischemia (under the supervision of Dr. Wanjun Li), written informed consent was obtained from all participants prior to sample collection, ensuring transparency and compliance with ethical standards. The collected samples were mixed with whole blood (10 mL per participant), utilized solely for in vitro investigations. No human samples were used for in vivo studies; all experiments with these samples were strictly in vitro.

### Synthesis of Te‐COF

4,4′,4′“,4′”‐(ethene‐1,1,2,2‐tetrayl) tetrabenzaldehyde and 4,4′‐ditellurodiphenylamine were dissolved in 1 mL of mesitylene, 1 mL of 1,4‐dioxane, and 0.6 mL of 6 m aqueous acetic acid in a 5 mL Schlenk test tube. After ultrasonic pulverization, the mixture was degassed by three freeze‐pump‐thaw cycles and flame‐sealed under vacuum. The sealed tube was then heated at 120 °C for 3 days. After cooling to room temperature, the resulting precipitate was isolated by filtration, washed repeatedly with tetrahydrofuran and acetone, and dried under vacuum to obtain the final product.

### Synthesis of Te‐COF@PEG600, Te‐COF@PEG2000, and Te‐COF@PEG6000

Te‐COF (10 mg) and either Hydrazide‐PEG600‐Hydrazide, Hydrazide‐PEG2000‐Hydrazide, or Hydrazide‐PEG6000‐Hydrazide (20 mg) were added to a tube containing 1 mL mesitylene and 1 mL 1,4‐dioxane. The mixture was subjected to ultrasonic pulverization for 2 h. Afterward, 0.6 mL of 6 m acetic acid aqueous solution was added, and ultrasonic pulverization was continued for 30 min. After three freeze‐pump‐thaw cycles, the mixture was dialyzed with deionized water (3 cycles of 4 L each) to eliminate any unpolymerized impurities. The final products, Te‐COF@PEG600, Te‐COF@PEG2000, and Te‐COF@PEG6000, were obtained by freeze‐drying.

### NBT Method for Measuring Glycated Serum Protein (GSP)

Serum samples from diabetic patients, mice, or rabbits were reacted with nitroblue tetrazolium (NBT) in an alkaline buffer (pH 10.8) at 37 °C for 15 min. GSP reduces NBT to a purple formazan product, and the absorbance was measured spectrophotometrically at 550 nm. Fresh serum was diluted, mixed with NBT reagent, incubated, and centrifuged to remove particulates. GSP concentration was calculated against a fructoselysine standard curve, following strict control of reagent freshness, incubation conditions, and exclusion of hemolyzed or lipemic samples (typical reference range: 1.08–2.79 mmol L^−1^).

### Thiobarbituric Acid (TBA) Method for Detecting Serum HbA1c

Detection involves acid hydrolysis of glycated hemoglobin to release 5‐hydroxymethylfurfural (5‐HMF), which subsequently reacts with TBA under high‐temperature conditions (e.g., 95 °C) to form a pink chromogen. Serum samples from diabetic patients, mice, or rabbits were mixed with a trichloroacetic acid reagent to precipitate proteins, followed by centrifugation to collect the supernatant. The supernatant was then heated with TBA, and the absorbance of the resulting compound was measured spectrophotometrically at 443 nm. HbA1c concentration was calculated using a standard curve or reference standards. Critical steps include precise control of hydrolysis time and temperature, inhibition of hemolysis, and calibration with quality controls. The method correlates HbA1c levels (typically 4–6%) with long‐term glycemic control.

### Diabetic Cardiomyopathy Model

Healthy 8‐week‐old male C57BL/6J mice and New Zealand rabbits were purchased from Nanjing GemPharmatech Co., LTD., and provided with normal water and feed during the adaptation period. Male C57BL/6J mice or New Zealand rabbits were fed a high‐fat, high‐sugar diet and housed under specific pathogen‐free conditions. Animals were randomly divided into Te‐COF@PEG2000 and model groups, and STZ (100 mg kg^−1^) was mixed with sodium citrate buffer (intraperitoneal injection) for two consecutive days. Under normal conditions, mice or rabbits with blood glucose levels ≥16.7 mm 4 weeks after the first injection of STZ were considered successfully diabetic. Diabetic animals were kept in a hyperglycemic state for four weeks.

### Detection of Serum‐Related Indexes

After static centrifugation, the supernatant of the mice blood was collected to get the serum. The levels of ALT and AST in each group were detected by an automatic biochemical analyzer (Mindray, China). According to the instructions of the kit, the levels of serum IL‐1 𝛽, IL‐6, TNF‐𝛼, and cTnI in mice and rabbits were detected by ELISA.

### Statistical Analysis

All analytical data were expressed as mean ± standard error of the mean. Differences between groups were analyzed using a one‐way analysis of variance (ANOVA) followed by a Dunnett t‐test. *p*‐Values: ^*^ *p* < 0.05, ^**^ *p* < 0.01, ^***^ *p* < 0.001, ^****^ *p* < 0.0001 ANOVA.

## Conflict of Interest

The authors declare no conflict of interest.

## Author Contributions

J.X., J.Z., and T.F. contributed equally to this work. All authors have given approval to the final version of the manuscript. G.Z., H.W., X.Z., and D.H. conceived and designed the project. J.X., J.Z., T.F., and J.W. carried out most of the experiments and analyzed the data. X.L., J.L., W.L., J.S., D.H., X.Z. performed part of the experiments. G.Z., J.X., and H.W. finalized the manuscript.

## Supporting information



Supporting Information

## Data Availability

The data that support the findings of this study are available from the corresponding author upon reasonable request.
