# Supplementary material for: Redox‐Responsive Tellurium‐Bridged Covalent Organic Frameworks/PEG Composites for Targeted Therapy of Diabetic Cardiomyopathy
Source: Adv Sci (Weinh). 2025 Nov 18;13(7):e09298. doi: 10.1002/advs.202509298 (PMC12866811; doi:10.1002/advs.202509298)
Supplement: Supplementary file 1 — Supporting Information [file ADVS-13-e09298-s001.docx]

**Supplemental information**

Redox-Responsive Tellurium-Bridged Covalent Organic Frameworks/PEG Composites for Targeted Therapy of Diabetic Cardiomyopathy

Jing Xue,^[a]^ Jialu Zhuang,^[b]^ Taotao Fan,*^[c]^ Songtao Tang,^[d]^ Junjun Wang,^[a]^ Xiaohui Liu,^[a]^ Jian Liu,^[e]^ Jin Shi^[a]^, Jiaji Li,^[a]^ Wanjun Li, ^[d]^ Danyou Hu,^[a]^ Xiaoqian Zhang,*^[d]^  Hua Wang,*^[b]^  Guiyang Zhang*^[a]^

[a] J. Xue, J. Wang, X. Liu, J. Shi, J. Li, D. Hu, G. Zhang

Department of Pharmacology

School of Pharmacy

Anhui Medical University

Hefei 230032, China

E-mail: guiyangzhang@ahmu.edu.cn

[b] J. Zhuang, H. Wang

Department of Oncology

The First Affiliated Hospital of Anhui Medical University, Inflammation and Immune-Mediated Diseases Laboratory of Anhui Province

Anhui Medical University

Hefei 230032, China

E-mail: [wanghua@ahmu.edu.cn](mailto:wanghua@ahmu.edu.cn)

[c] T. Fan

Department of Functional Experiment Center

School of Basic Medical Sciences

Anhui Medical University

Hefei 230032, China

[d] S. Tang, W. Li, X. Zhang

Department of Endocrinology

First Affiliated Hospital

Anhui Medical University

Hefei 230032, China

[e] J. Liu

College of Chemical and Pharmaceutical Engineering

Hefei Normal University

Hefei 230601, China

**Materials.** Mesitylene (98%), 1,4-dioxane (99%) and ethyl acetate (99.5%) were purchased from Aladdin, China. 4,4',4'',4'''-(ethene-1,1,2,2-tetrayl) tetrabenzaldehyde, 4,4'-ditellurodiphenylamine, Hydrazide-PEG600-Hydrazide, Hydrazide-PEG2000-Hydrazide, Hydrazide-PEG6000-Hydrazide, Tetrahydrofuran, DMSO, sodium citrate, and acetone were purchased from Tensus Biotech, China. GSP test kit, GHb test kit, Glu test kit, TP test kit, albumin test kit, ApoA1 test kit, ApoB test kit, HDL-C test kit, LDL-C test kit, TC test kit, TG test kit, ALT test kit and AST test kit were purchased from Mindray, China. Elisa kits for IL-1𝛽, IL-6, TNF-𝛼, and other inflammatory factors were purchased from ELK Biotechnology, China. For immunofluorescence staining, F4/80, CD206, TUNEL, ROS and other antibodies were purchased from CST, USA.

**Modeling and Structure Optimization of Te-COF.** The crystal modeling of Te-COF structure was carried out using Materials Studio. According to the distance between the vertex centers of Te COF, the cell parameters were estimated, which was observed in similar materials. Initially, these structures were identified as overlap ping structures. To optimize the structure, a geometric optimization method combining energy minimization and elemental parameter optimization was employed. The calculation of simulated powder diffraction pattern and Pawley refinement were performed with the material studio reflection plus module using Bragg-brentano geometry. The observed diffraction patterns were subtracted by polynomial background and then refined by Pawley. Pseudo-Voight peak shape function was used to refine the peak shape, and Berar-Baldinozzi function was used to correct the asymmetry. The crystal size was estimated by the Le Bail method, and experimental PXRD data were fitted using the Pawley refinement approach.

**Powder X-Ray Diffraction.** PXRD data were acquired on a D8 Advancex X-ray diffractometer using a step scan rate of 0.02°, an exposure time of 2 seconds, and a scan range of 1°-50° for optimal counting statistics.

**Nitrogen Adsorption/Desorption Analysis.** Nitrogen adsorption was measured with a Micromeritics Samsung II 3020 (Micromeritics, Norcross, Ga.) at 77 K. Brunauer–Emmett–Teller (BET) equation was used to estimate the specific surface area. In the second linear region of the N_2_ isotherm, the internal and external surface areas of the graphs were determined according to the Harkins and Jura equation (P/P_0_ =0.26-1.0).

**Thermogravimetric Analysis (TGA).** TGA was performed on a TAInstrumentsQ-500 thermogravimetric analyzer by placing the samples in an alumina canister under an atmosphere of continuous flow of nitrogen. Samples (Te-COF, Te-COF@PEG600, Te-COF@PEG2000 or Te-COF@PEG6000) were first preheated at 200 °C for 2 hours to remove residual solvent molecules After cooling to room temperature, the samples were analyzed with a heating rate of 10 °C min⁻¹ under continuous nitrogen flow.

**Cytotoxicity Study of Te-COF@PEG600, Te-COF@PEG2000 and Te-COF@PEG6000 *In Vitro*.** Myocardial macrophages (RAW264.7 cell line) of logarithmic growth stage were collected, and the cell suspension was adjusted to an appropriate concentration. Cells were seeded into 96-well plates and incubated at 37 °C to allow adherence. Te-COF@PEG600, Te-COF@PEG2000, or Te-COF@PEG6000 with the same concentration gradient was then added, and the control group was added with the same amount of PBS. After 48 hours of incubation, cells were washed and incubated with 3-(4,5)-dimethylthiazolyl (-z-y1) −3,5-diphenyltetrazolium (MTT) solution. Following further washing, the resulting purple formazan crystals were dissolved in DMSO, and the absorbance was measured at 570 nm.

**Hemolysis Test.** Eyeball blood of the C57BL/6J mice was collected in an anticoagulant tube containing sodium citrate. After centrifugation at 3000 rpm for 15 minutes, 20 μL of the blood cells were extracted and mixed with the following solutions: 1 mL of deionized water (DW), 1 mL of PBS, 50 μL of Te-COF@PEG2000 + 1 mL of PBS. The mixture was incubated at 37 °C for 4 hours and centrifuged again at 3000 rpm for 15 minutes. Subsequently, 100 μL of the supernatant solution was transferred to a 96-well plate, and the absorbance at 542 nm was measured using a multifunctional enzymoscope. The hemolysis rate was calculated using the following formula: hemolysis rate (%) = [OD (sample group) − OD (PBS group)]/[(OD (pure water group) − OD (PBS group)]× 100%. Different volumes of Te-COF@PEG2000 (25, 50, 75, 100 μL) were each mixed with 1 mL of PBS to determine the hemolysis rate of gels with different concentrations.

**Masson Staining.** Tissue sections were heated at 60 °C for 2 hours using a thermostatted heater and dewaxed (xylene I 20 minutes → xylene II 20 minutes → anhydrous ethanol I 10 minutes → anhydrous ethanol II 10 minutes → 90% ethanol 10 minutes → 80% ethanol 5 minutes → 70% ethanol 5 minutes → distilled water 5 minutes), tissue sections were immersed in Bouin (A) solution, heated to 37 °C with a thermostatted heater for 2 hours, dyed mordant, and rinsed with tap water until the tissue sections turned yellow.

**Sirius Red Staining.** Tissues were fixed in 10% formalin, embedded in paraffin, and sectioned (4–5 μm). Sections were deparaffinized with xylene, rehydrated through graded ethanol, and stained with 0.1% Sirius Red in saturated picric acid for 1 hour. After differentiation with 0.5% acetic acid, sections were dehydrated, cleared in xylene, and mounted for imaging.

**Hematoxylin and Eosin (H&E) Staining.** Myocardial tissues from each group of mice or rabbits were collected, embedded in paraffin, and sectioned. Following embedding, tissue dehydration was performed through graded xylene and anhydrous ethanol series. Subsequently, sequential staining was performed using hematoxylin and eosin. After sealing, myocardial tissue structure and cell necrosis were observed. The quantification of the myocardial necrosis area was conducted using Image J software.

**TTC Staining.** Fresh tissue slices (1–2 mm thick) were incubated in 1% TTC solution at 37 °C for 15–30 minutes in the dark. The staining was stopped by rinsing with PBS, followed by fixation in 4% paraformaldehyde. Viable tissue stained red due to mitochondrial dehydrogenase activity, while necrotic areas remain unstained. The slices were observed under a microscope or photographed for analysis. Incubation time was adjusted based on tissue type and samples were stored in the dark to preserve staining. The slices were placed in order and photographed against a black background. The tail side of each slice was selected for image analysis using ImageJ (version 1.61). The infarct volume of each layer was measured as the product of the infarct area and thickness of the layer. The total infarct volume was obtained by summing the volumes of all layers.

**Statistical Analysis.** All analytical data were expressed as mean ± standard error of the mean. Differences between groups were analyzed using a one-way analysis of variance (ANOVA) followed by Dunnett-t-test. *p*-Values: **p* < 0.05, ***p* < 0.01, ****p* < 0.001, *****p* < 0.0001 ANOVA.

**Table of Contents**

**Figure S1:** Experimental PXRD pattern of Te-COF with Pawley refinement and simulated profile

**Figure S2:** Solid-state ¹³C CP-MAS NMR spectrum of Te-COF

**Figure S3:** Zeta potential of Te-COF and Te-COF@PEG composites

**Figure S4:** *In vitro* optimization of Te-COF@PEG composites

**Figure S5:** *In vivo* biosafety evaluation of Te-COF@PEG composites *via* serum biomarker detection

**Figure S6:** F4/80 fluorescence staining of rabbit myocardial tissue

**Figure S7:** CD206 fluorescence staining of rabbit myocardial tissue

**Figure S8:** TUNEL fluorescence staining of rabbit myocardial tissue

**Figure S9:** ROS fluorescence staining of rabbit myocardial tissue

**Figure S10:** Fluorescence imaging and fluorescence intensity characterizations


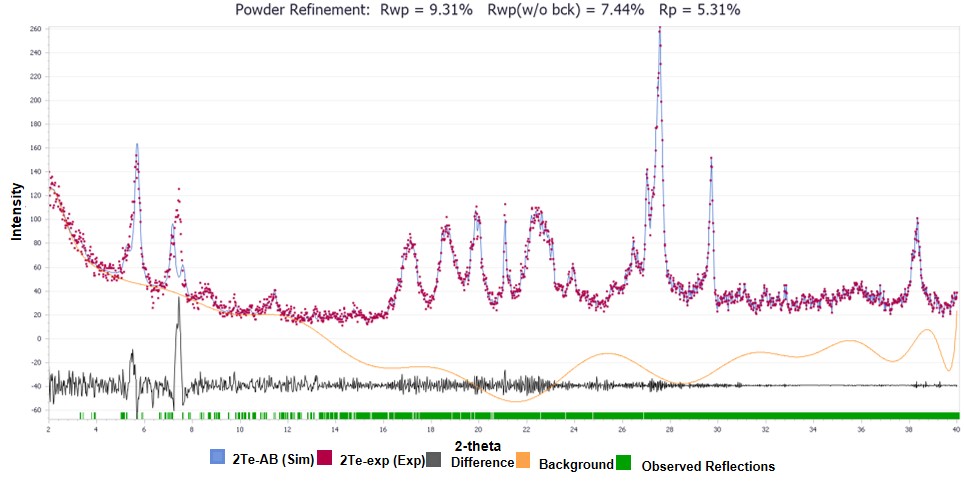


**Figure S1.** Experimental PXRD patterm of Te-COF with Pawley refinement and simulated profle.


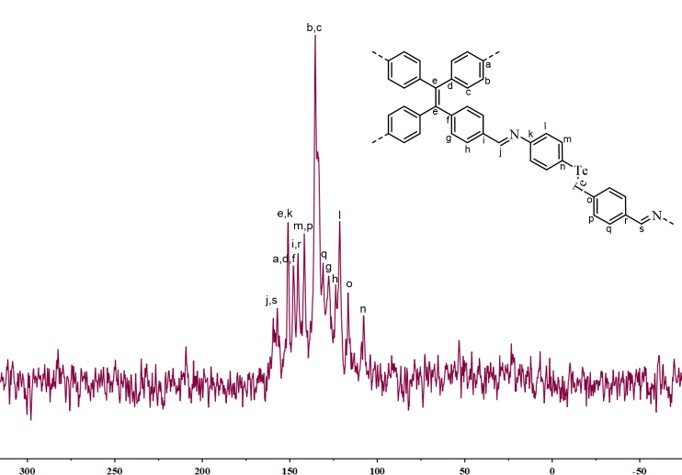


**Figure S2.** Solid-state ^13^C CP-MAS NMR spectrum of Te-COF.


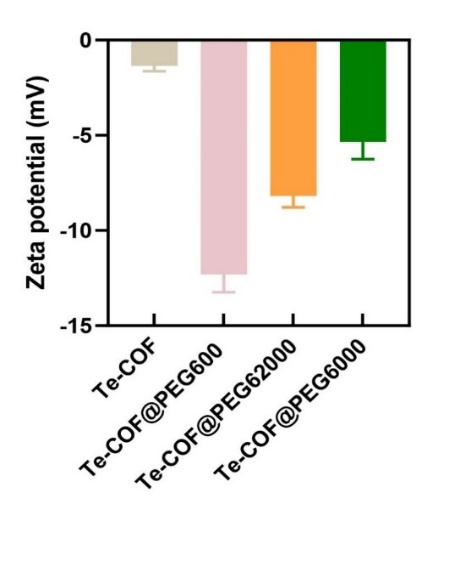


**Figure S3**. The Zeta potential of Te-COF and Te-COF@PEG composites.


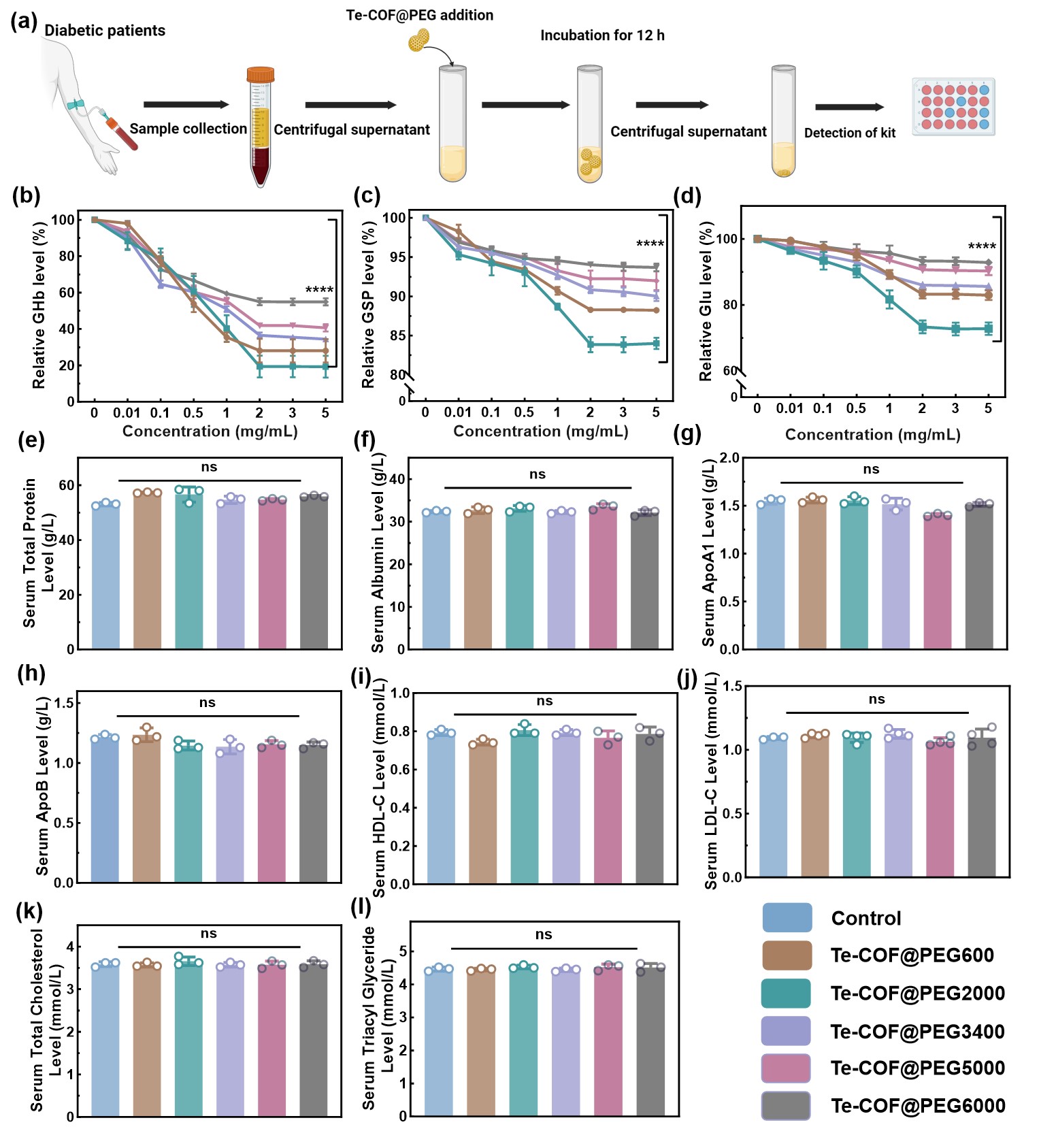


**Figure S4**. *In vitro* optimization of Te-COF@PEG composites. Te-COF@PEG composite was co-incubated with the serum samples of diabetic patients, and changes in the relative levels of the total GSP (a), glycosylated HbA1c (b), blood Glu (c), total protein (d), ALB (e), ApoA1 (f), ApoB (g), HDL-C (h), LDL-C (i), TC (j) and TG (k) were detected after the intervention of the Te-COF@PEG composites. Data represent mean ± SD (*n* = 3); *p*-Values: *****p* < 0.0001.


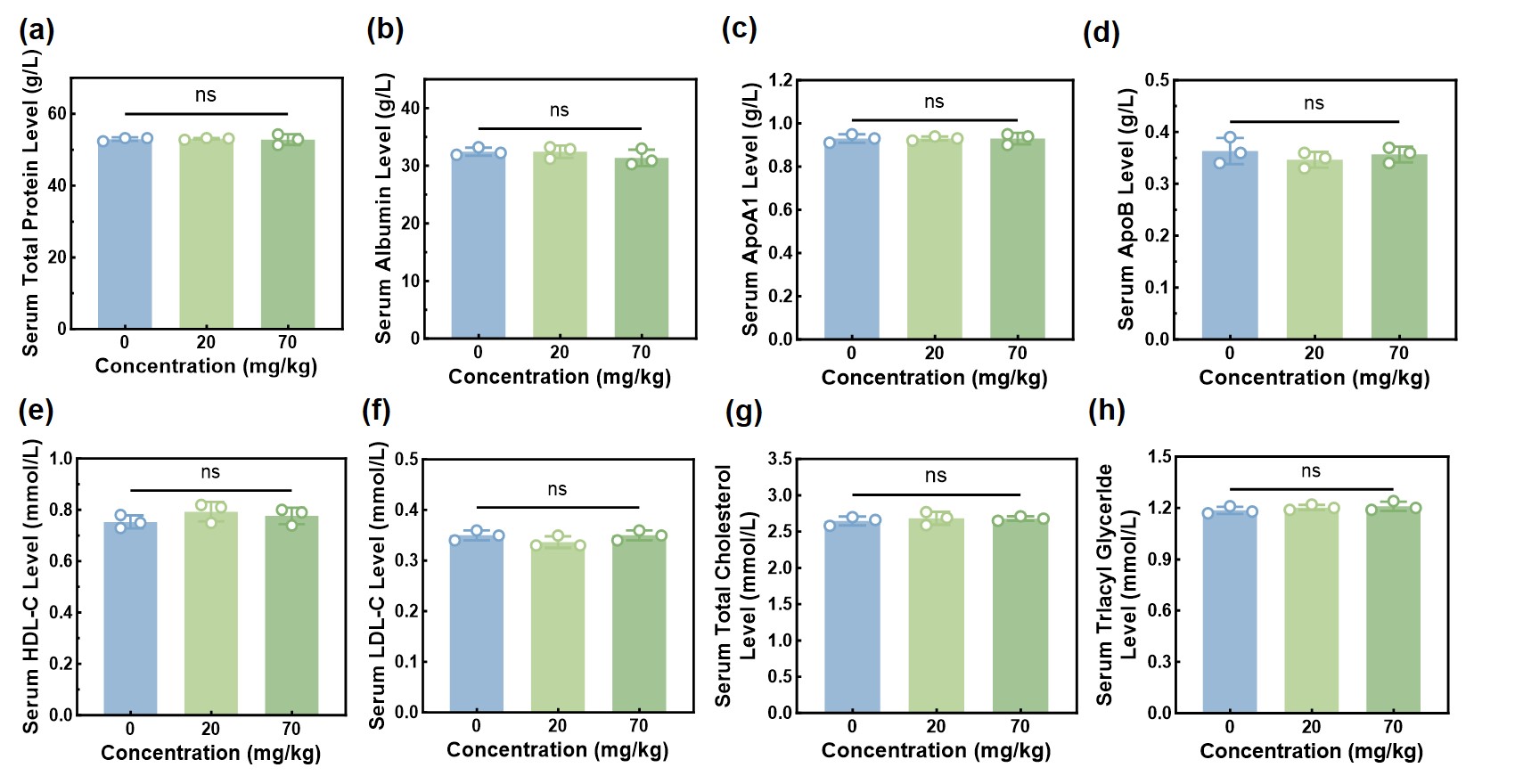


**Figure S5**. *In vivo* biosafety evaluation of Te-COF@PEG composites *via* serum biomarker detection. (a) Serum Total Protein Level, (b) Serum Albumin Level, (c) Serum ApoA1 Level, (d) Serum ApoB Level, (e) Serum HDL-C Level, (f) Serum LDL-C Level, (g) Serum Total Cholesterol Level, and (h) Serum Triacyl Glyceride Level were measured in experimental groups treated with different concentrations of Te-COF@PEG composites. All data are presented as mean ± SD (*n* = 3), and “ns” indicates no significant difference, demonstrating that the composite has no obvious adverse effects on these serum biomarkers at the tested concentrations*.*


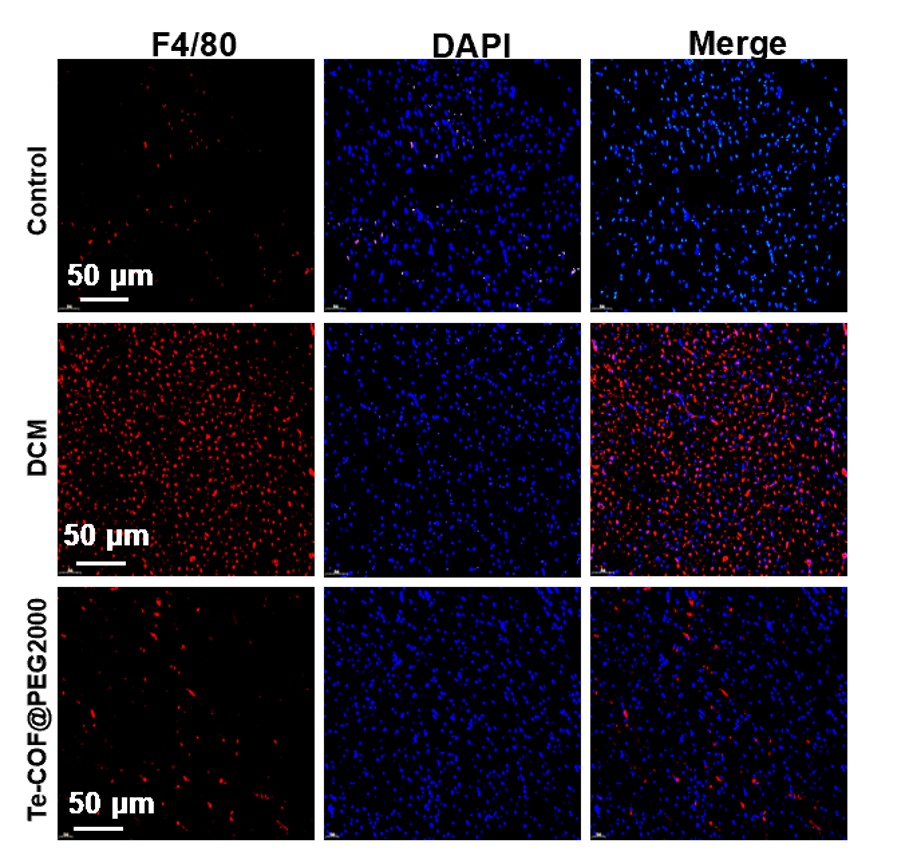


**Figure S6.** F4/80 fluorescence staining of myocardial tissue of rabbits (*n* = 4). Scale bar, 50 μm.


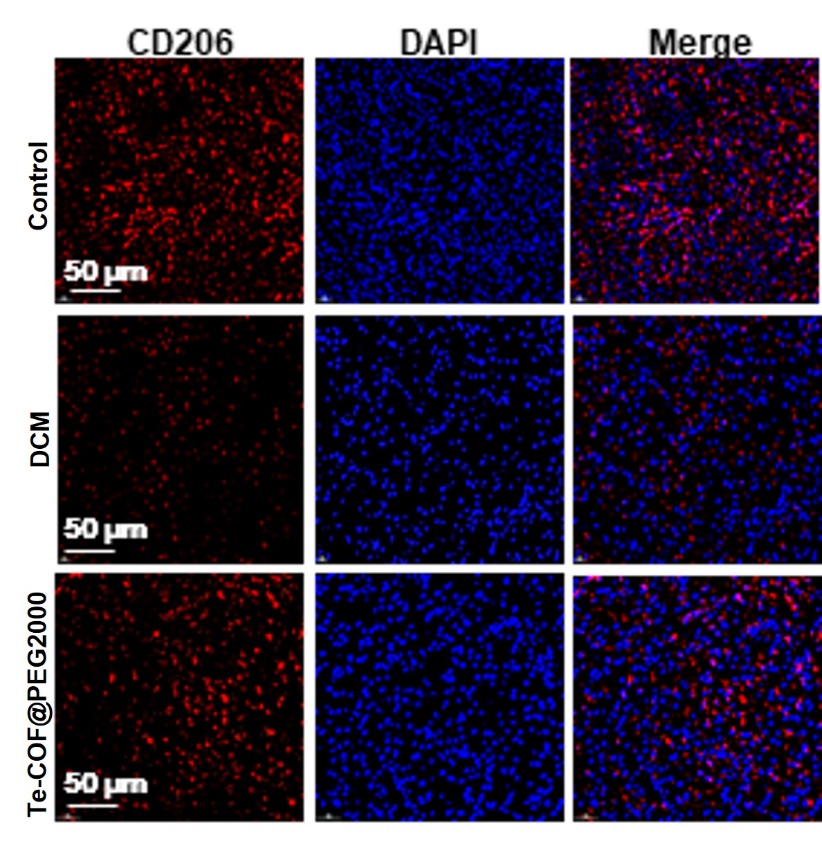


**Figure S7.** CD206 fluorescence staining of myocardial tissue of rabbits (*n* = 4). Scale bar, 50 μm.


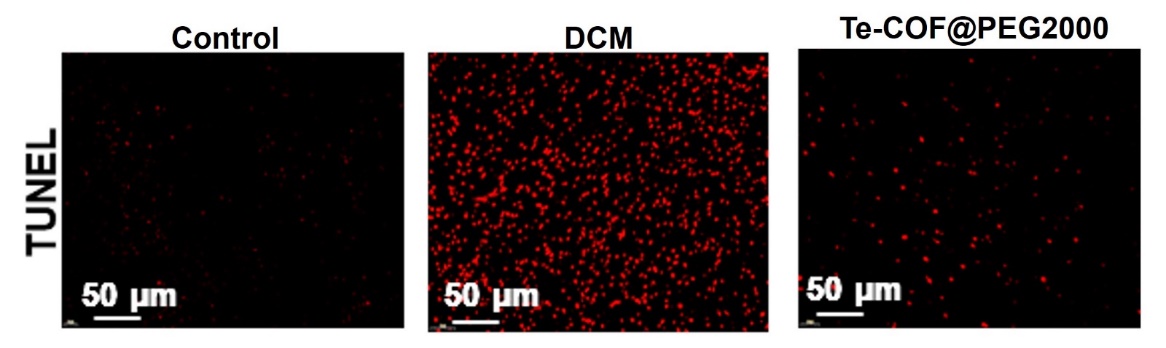


**Figure S8.** TUNEL fluorescence staining of myocardial tissue of rabbits (*n* = 4). Scale bar, 50 μm.

**
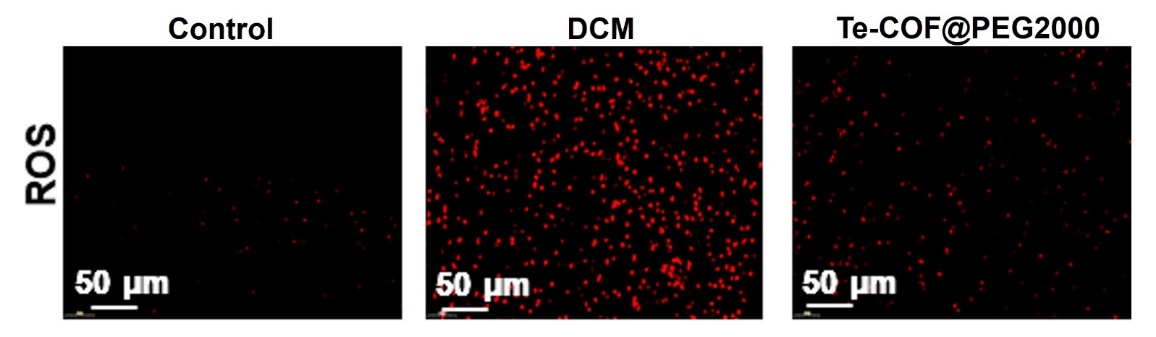
**

**Figure S9.** ROS fluorescence staining of myocardial tissue of rabbits (*n* = 4). Scale bar, 50 μm.


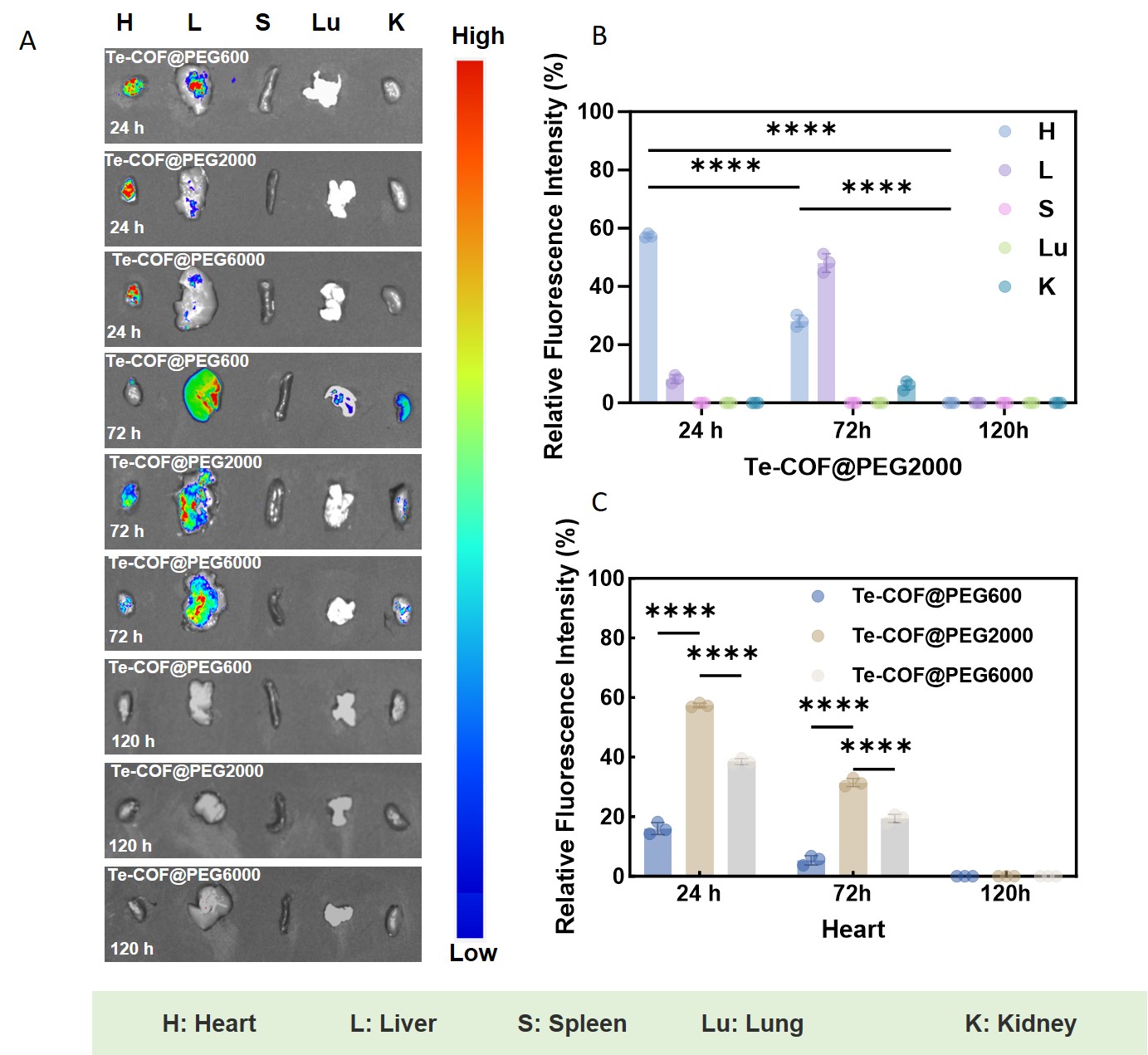


**Figure S10. Fluorescence imaging and fluorescence intensity characterizations. A.** Ex vivo fluorescence images (ex: 405 nm) of Te-COFs modified with different PEG molecular weights (Te-COF@PEG600, Te-COF@PEG2000, Te-COF@PEG6000) in the heart (H), liver (L), spleen (S), lung (Lu), and kidney (K) at 24 h, 72 h, and 120 h. **B.** Statistical analysis of the relative fluorescence intensity of Te-COF@PEG2000 in the heart, liver, spleen, lung, and kidney at 24 h, 72 h, and 120 h. **C.** Statistical analysis of the relative fluorescence intensity of Te-COFs modified with different PEG molecular weights (Te-COF@PEG600, Te-COF@PEG2000, Te-COF@PEG6000) in the heart at 24 h, 72 h, and 120 h. **** indicates extremely significant differences between groups. Data represent mean ± SD (*n* = 3); *****p* < *0.0001*.
